# Teguest Guerma: making a difference in public health

**DOI:** 10.2471/BLT.19.030919

**Published:** 2019-09-01

## Abstract

Teguest Guerma talks to Gary Humphreys about her career in public health, and her commitment to training midwives in Ethiopia.

Q: How did you become an infectious diseases expert?

A: I blame my mother! You see, she really wanted to become a nurse, but her father married her off when she was 17, so she promised herself that all her daughters would become medical doctors. And she made sure it happened. She really brainwashed us! She even showed us films with heroic doctors in them. Of course, when I became a doctor myself I found that it was not like the films at all, and I warned my younger sisters, but they still went ahead. So now we have an infectious disease specialist, an ophthalmologist and a psychiatrist in the family.

Q: You grew up in Ethiopia but ended up practising medicine in Burundi. How did that happen?

A: It’s a long story. I started my medical studies in Reims in France where I had a scholarship, but I was forced to pursue dental medicine because of their quota system. I did that for three months, but it was not my passion, so I looked around for other options and in the end completed my medical studies at the University of Dakar in Senegal, where I became an infectious diseases specialist. It was in Senegal that I met my husband. He was from Burundi, which is where we went to live and where I first started to practise medicine. It was also in Burundi that I was first confronted with HIV.

Q: I understand you diagnosed some of the first cases in Burundi. Can you talk about that?

A: It was in the mid-1980s and I was head of internal medicine at the public hospital in Bujumbura. People started to come into the hospital with the symptoms of acquired immunodeficiency syndrome (AIDS), which I recognized from early descriptions of cases in the United States of America. I had never seen anyone with AIDS and only a couple of cases had been reported in Burundi. So, it was a shock and there was real reluctance to talk about it.

Q: Is it true the authorities tried to cover it up?

A: Absolutely. The first time it really hit me that there was an actual cover-up going on was when Daniel Tarantola, the first WHO deputy director of the Global Programme of AIDS, came to visit the hospital. I was asked to show him around and I showed him all the HIV patients in my ward, but when we went to the other wards all the patients had disappeared. I found out afterwards that my colleagues had been told to hide their AIDS cases. Nobody had said anything to me because they knew that I would have said no.

“My colleagues had been told to hide their AIDS cases.”

Q: Why was there such secrecy?

A: The government in Burundi thought that it was going to be an outbreak that would just blow over. And they said that if we talked about it openly it would have an impact on the economy. So, it was a real taboo. All my colleagues were sending me their patients and I had the job of telling these people that they were infected. I was trying to persuade them to protect their partners. It was not easy. Remember, a diagnosis of HIV infection was a virtual death sentence at that time. Also, because we couldn’t talk about it, we were doing absolutely nothing on the prevention side. In the end, the word got out about the situation. Data were leaked to the press by a colleague of mine, who was subsequently deported within 24 hours. The security service people came to my office, smashed everything, took all my documents and threatened to put me in prison. At that time, I knew that I was going to have to leave.

Q: Is that what you did?

A: Yes. I got in touch with Daniel Tarantola. He had been very encouraging about the work I was doing there, but I didn’t expect to get a job. However, I wrote to him anyway and three weeks later he responded saying that he could offer me a short-term consultancy. He sent me to the Republic of Congo to develop a short-term HIV plan. Then I was recruited as part of Jonathan Mann’s team working on the Global Programme of AIDS in Geneva, a real dream team, as it turned out. Working on it gave me the commitment and dedication needed for the following 21 years at WHO. My job was to assist African countries in developing their short-term plans on HIV. I was the one who developed the first HIV sentinel surveillance systems.

Q: You are most associated with the 3 by 5 initiative that aimed to give 3 million people in need in low- and middle-income countries access to HIV treatment by 2005. How did you became involved in that?

A: I was in New York representing the WHO Regional Office for Africa and advocating around different health issues in the United Nations at the time, having spent several years working on HIV in Africa and Asia. I got a call from Dr Jim Kim Yong, who had taken over the 3 by 5 initiative. He was new to WHO and Africa, and he asked me if I would help him. I said, “Do you just want an African woman to decorate your office like a flower or do you want someone to help you make a difference?” As it turned out he wanted to make a difference and we worked very well together.

Q: What do you consider the main achievements of the 3 by 5 initiative?

A: The most obvious benefit was helping people from developing countries access generic antiretroviral drugs at affordable prices, which is to say at US$ 140 per year instead of US$ 15 000. But the initiative also informed the development of WHO’s public health approach for antiretroviral therapy and was instrumental in establishing a drug prequalification department in WHO. Ultimately it led to people getting access to drugs free at the point of delivery, which was no small achievement given the pressure WHO was under from different parties who had an interest in maintaining the status quo. Along with the work I did at country and regional level, I consider my work on 3 by 5 to be my biggest contribution in the fight against HIV.

Q: You also led the revision of the new HIV treatment guidelines in 2010. Can you explain 

the significance of those guidelines?

A: At that time antiretroviral treatment was being started very late, which is to say at a CD4 count of 200 cells per μL. So, the first recommendation was to start treatment for all HIV-infected individuals at 350 CD4 cells per μL or less. This meant treating a lot more people, but earlier treatment had been shown to greatly improve clinical outcomes. The guidelines also recommended moving away from certain toxic medicines like stavudine and replacing them with newer and more effective – but more expensive medicines – such as tenofovir. There was evidence that these changes would increase survival of AIDS patients in low- and middle-income countries. As an evidence-based organization, WHO was obliged to revise its guidelines despite the pressure from some donors who were unwilling to spend more money on treatment. Another important change was the test-and-treat approach, which put the emphasis on identifying people in need of antiretroviral treatment instead of relying on people to come forward voluntarily for diagnosis and treatment. These days test-and-treat is taken for granted, but at the time there was a lot of discussion about different issues, including the cost implications.

“A key objective is to train young women… [to] go back to their communities and help their sisters giving birth.”

Q: Despite these achievements you decided to take early retirement in 2010.

A: And the day I retired I was head-hunted for the position of Director-General of Amref Health Africa. I became the first African woman to head the organization. It was quite a change for me. For the first time I felt that I was really making a difference to the health of the communities we worked with.

Q: Did you continue your focus on HIV?

A: Not at all. My first major campaign was called Stand up for African Mothers, it was committed to mobilizing support to train 15 000 midwives by 2015 in Africa. The campaign really brought home to me again the importance of skilled midwives in the reduction of maternal and infant mortality in Africa.

Q: You left Amref in 2015 to establish a school of midwifery. Can you talk about that?

A: In the 42 years I lived outside my country I had a dream. I wanted to come back and support women in Ethiopia. That is why I decided to establish a midwifery college in the centre of Addis Ababa on my family’s land and with my retirement fund. It is a Bachelor of Science College that is accredited by the government. We can accommodate 240 students and this year took in our first 40. We plan to take another 80 next year and so on until we reach capacity. We want to become a centre of excellence to drive health workforce capacity development in the rest of the country. A key objective is to train young women from rural areas; to have them come to Addis, learn these skills and then go back to their communities and help their sisters giving birth. The students make a commitment to serve their community for two years after they graduate. Many of our first students are from the Afar and Somali Regions where the maternal death rate is relatively high.

Q: It sounds like you have found somewhere else to make a difference.

A: I believe I have. And it is very personal for me since I almost died when my mother was giving birth. The doctors couldn’t get me out and I was delivered by C-section. They put me to one side and said, “Let her die and let’s save the life of the mother”. I didn’t die, of course, but I almost certainly would have if I had been born poor in a rural area instead of having relatively rich parents in Addis Ababa. I have often thought about that since and want to do something for women less fortunate than myself.

